# Stretchable Full‐Color Phosphorescent PVA‐Based Ionogels for Multimodal Sensing‐Visual Integration Applications

**DOI:** 10.1002/advs.202411229

**Published:** 2024-12-12

**Authors:** Xuefeng Wei, Zexi Gou, Jianting Ye, L. H. Shi, Jianwei Zhao, Lei Yang, Linbo Zhang, Kun Zhang, Ruonan Jia

**Affiliations:** ^1^ Sichuan Provincial People's Hospital School of Medicine University of Electronic Science and Technology of China Chengdu 610072 China; ^2^ College of Materials and Chemistry & Chemical Engineering Chengdu University of Technology Chengdu 610059 China; ^3^ National Engineering Research Center of Electromagnetic Radiation Control Materials Key Laboratory of Multi‐spectral Absorbing Materials and Structures of Ministry of Education University of Electronic Science and Technology of China Chengdu 610072 China; ^4^ Shenzhen HUASUAN Technology Co., Ltd. Shenzhen 518107 China

**Keywords:** ionogels, optoelectronics, phosphorescence, PVA, stretchable

## Abstract

Exploring ionogels with superior conductivity, mechanical properties, and long‐lasting room temperature phosphorescence (RTP) offers considerable potential for new‐generation optoelectronics. However, reports on ionogels remain limited owing to the contradiction between the flexibility required for stretching and the rigidity necessary for RTP and load‐bearing within the same ionogels. Here, a facile strategy is reported to enhance the toughness and extend the RTP of ionogels by salting‐out‐induced microphase separation, which results in the formation of an IL‐rich phase (soft) for stretching and ionic conduction and a polymer‐rich phase (stiff) for energy dissipation and clustering‐triggered phosphorescence. The obtained ionogels exhibit high stretchability (≈400% strain), toughness (≈∼20 MJ m^−3^), ionic conductivity (8.4 mS cm^−1^), and ultralong afterglow lifetime (112.4 ms). This strategy is applicable to chromophores with color‐tunable phosphorescence. By leveraging observable full‐color RTP and real‐time electrical signals in response to diverse stimuli (i.e., stretching and pressing), an intelligent grasping strategy is developed for robust hand pose reconstruction. In addition, a tactile‐visual fusion recognition keyboard is created with dual functionality of information encryption and signal transmission. The ease of fabrication, wide tunability, and multifunctionality will help expand the scope of ionogels for smart devices.

## Graphical abstract

Salting‐out‐induced microphase separation, instead of sophisticated synthesis, is adopted to fabricate IL‐rich phase and polymer‐rich phase, which synergistically enhance the stretchability, ionic conductivity, RTP, and mechanical properties of ionogels. A novel human‐machine interaction mode is developed by integrating visually high signal‐to‐noise ratio full‐color RTP with mechano‐transduction ability in stretchable PVA‐based ionogels.

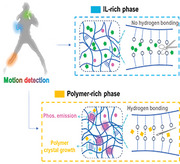

## 1 Introduction

Stretchable ultra‐long room‐temperature phosphorescence (RTP) displays integrated with skin‐like high‐sensitivity electronic sensing are highly desirable in the development of optoelectronics for versatile applications such as soft robots, intelligent monitoring, and wireless communication.^[^ ^1^ ^]^ Such a tactile–visual fusion integration strategy would facilitate more intelligent interaction with both users and environments.^[^ ^2^ ^]^ However, traditional bioinspired artificial skins and intelligent systems rely heavily on electrical signals and seldom consider the visual perception necessary for interaction in complex and dynamic environments. Room‐temperature phosphorescence (RTP) materials have attracted increasing attention owing to their advantages over fluorescence materials, such as high signal‐to‐noise ratios, larger Stokes shifts, and naked‐eye‐observable afterglow.^[^ ^3^ ^]^ Two crucial principles must be considered to obtain RTP‐based multimodal sensing‐visual integration materials: a) sustainability of RTP emission during mechanical deformation as artificial skin; b) ability to convert applied touch, pressure, and deformation into discernible electrical signals.

Owing to their inherent functional complementarity and material compatibility, ionogels—polymer networks swelled with ionic liquids (ILs)—offer considerable promise for wearable electronics.^[^ ^4^ ^]^ Ionogels that encompass substantial quantities of solvent typically exhibit elasticity.^[^ ^5^ ^]^ Nonetheless, the development of organic RTP materials necessitates the suppression of the non‐radiative decay of triplet excitons through the establishment of a rigid environment,^[^ ^6^ ^]^ in sharp contrast with the superior mobility and reconfiguration capacity of solvents and molecular chains in ionogels. The contradiction between the photophysical mechanism of RTP and the flexible nature of the ionogels poses a challenge to the development of stretchable and long‐lived RTP ionogels. Moreover, most ionogels exhibit weak mechanical properties, such as low tensile strength (<1 MPa) and toughness (≈1000 J m^−2^).^[^ ^7^ ^]^ In addition, most ionogels tend to sacrifice their electronic performance to obtain useful mechanical properties.^[^ ^8^ ^]^ Thus, we aim to develop a method to endow ionogels with an ultralong RTP performance while simultaneously achieving excellent mechanical properties and conductivity.

Considering the balance between stiffness and softness in the same ionogel is a key issue, multiphase engineering has recently been investigated as a facile platform for combining different components with multiple functions in a material.^[^ ^9^ ^]^ Atom‐transfer radical polymerization and reversible deactivation radical polymerization have been utilized to fabricate hard‐soft microphase‐separated structures through block copolymer self‐assembly.^[^ ^10^ ^]^ Instead of sophisticated synthesis, we hypothesized that the well‐designed arrangement and aggregation of molecular chains in ionogels could regulate the solvation and free volume of molecular chains to form gel‐like domains (IL‐rich phase) and rigid domains (polymer‐rich phase), respectively.

The Hofmeister effect is the specific effect of salting‐out ions to expel water molecules from the space between molecular chains, accompanied by the formation of hydrogen bonds and aggregation of polymer chains, which is well established in hydrogels.^[^ ^11^ ^]^ In this study, we found that salting‐out ions could also sufficiently interact with the hydroxyl bonds of the polymer to push the ionic liquid (IL) past its gel phase boundary, leading to the aggregation of molecular chains and subsequent microphase‐separated morphology in the ionogels. In the IL‐rich phase, ionic liquids screen ionic bonds and form a few hydrogen bonds, building highly stretchable networks and providing pathways for effective charge conduction. In contrast, the polymer‐rich phase with multiple hydrogen bonds is no longer a gel but is stiff, which limits non‐radiative deactivation and enhances RTP performance. Moreover, a polymer‐rich phase with abundant unsaturated bonds may exhibit strong non‐traditional phosphorescence in the aggregated state, i.e., clustering‐triggered emission (CTE).^[^ ^12^ ^]^ The salting‐out process induces phase separation and introduces counter ions (Na^+^) as additional transport ions to enhance the conductivity of the ionogels. Hence, the synergistic effect of these phases enables the formation of PVA‐based ionogels with superior conductivity, mechanical properties, and ultralong RTP; however, this approach has rarely been reported. Notably, this approach eliminates the need for laborious processing (e.g., multistep synthesis and solvent exchange), thus providing a practical and simple method to develop multifunctional ionogels.

## 2 Results

### 2.1 Fabrication and Characterization of Polyvinyl Alcohol/Polyacrylamide (PVA/PAM) Ionogels

**Figure 1:**
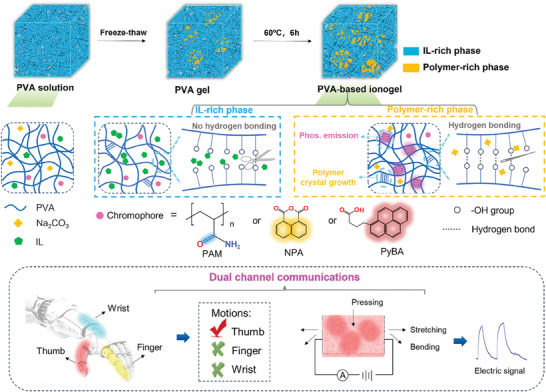
Schematics of stretchable RTP PVA‐based ionogels and potential applications.

As shown in **Figure** **1**, polyvinyl alcohol (PVA) is selected as the polymer matrix. Considering the rich non‐traditional luminogens (C═O and N─H groups), polyacrylamide (PAM) is introduced to further prove aggregation and microphase separation in ionogels based on its unique CTE phenomenon. The PVA gel was initially pre‐formed using the conventional freeze‐thaw method, during which small crystallites emerged and served as cross‐linking points.^[^ ^13^ ^]^ Subsequently, PVA/PAM ionogels encompassing both the polymer‐ and IL‐rich domains were obtained after being maintained at 60 °C for 6 h. This process entails the rearrangement and aggregation of molecular segments and aids in eliminating auxiliary solvents. To understand the influence of salting‐out ions on ionogel performance, we fabricated a series of PVA/PAM ionogels with the nomenclature PVA/PAM‐NaIL x, where x represents the concentration of CO_3_^2−^ in the ionogel (**Table** **1**). PVA/PAM ionogels without salting‐out ions (PVA/PAM‐IL) and PVA/PAM films without IL (PVA/PAM‐Na) were used as control groups. In our preliminary work, we explored the effects of different anions on the crystallinity and phosphorescence properties of PVA/PAM ionogels. The results indicated that CO_3_^2−^ more readily induced the aggregation and crystallization of molecular chains; correspondingly, the longest phosphorescence lifetime was observed (Figure S1, Supporting Information). Therefore, CO_3_^2−^ was selected as the model salting‐out ion in this study. Owing to their high ionic strengths, ILs with imidazolium cations have been utilized as solvents.

**Table 1:** Different formulations of PVA/PAM ionogels ant their corresponding mechanical and RTP properties.

Name	PVA wt.%	PAM wt.%	Na_2_CO_3_/PVA ratio	IL/PVA ratio	Strength [MPa]	Strain at break [%]	Toughness [MJ m^−3^]	Phos. lifetime [ms]
PVA/PAM‐IL	10	5	0	15/16	4.86	281.5	8.99	56.9
PVA/PAM‐NaIL 1	10	5	1/15	15/16	5.70	293.4	9.74	61.4
PVA/PAM‐NaIL 2	10	5	1/5	15/16	9.14	425.3	20.36	112.4
PVA/PAM‐NaIL 3	10	5	1/3	15/16	5.46	342.5	10.97	79.8
PVA/PAM‐Na	10	5	1/3	0	7.81	55.2	3.2	46.1

**Figure 2:**
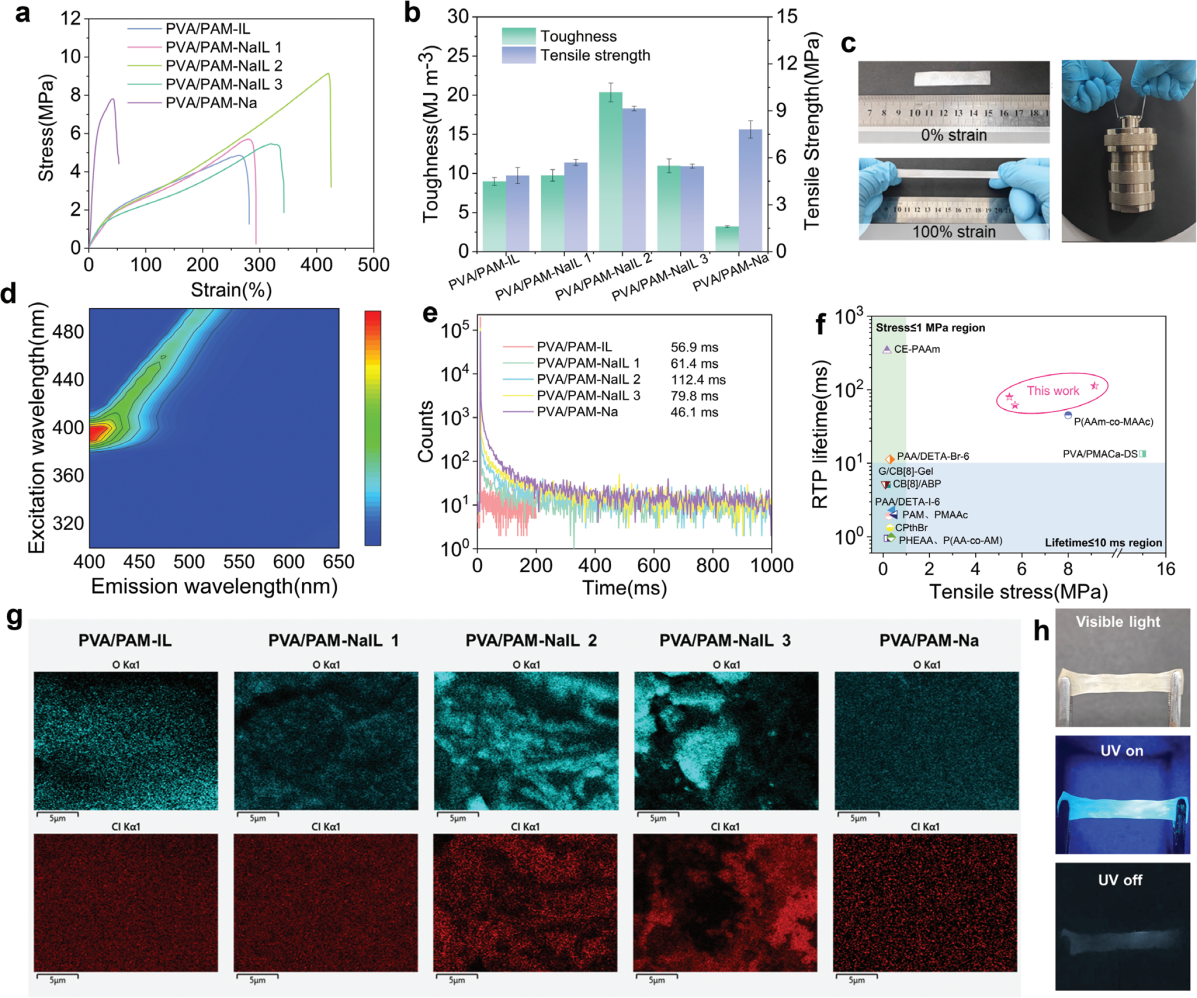
Structure, mechanical, and photophysical properties of PVA/PAM ionogels. a) Stress–strain curves of PVA/PAM‐IL, PVA/PAM‐NaIL and PVA/PAM‐Na. b) Tensile stress and calculated toughness of PVA/PAM‐IL, PVA/PAM‐NaIL and PVA/PAM‐Na. Error bars represent mean ± standard deviation (*n* = 3). c) Photograph of PVA/PAM‐NaIL 2 ionogels being stretched to 200% and lifted with heavy loads. d) Excitation fluorescence mapping of PVA/PAM‐NaIL 2 ionogel. e) Lifetime decay curves of PVA/PAM‐IL, PVA/PAM‐NaIL, and PVA/PAM‐Na. f) Comparison of the RTP performance of the PVA/PAM‐NaIL ionogels in this article with other recently reported works. g) EDS element mapping of PVA/PAM‐IL, PVA/PAM‐NaIL, and PVA/PAM‐Na. h) Photographs of fluorescent and phosphorescent PVA/PAM‐NaIL 2 in a stretched state.

Stress–strain curves were recorded to characterize the mechanical properties of the PVA/PAM ionogels. All PVA/PAM‐NaIL ionogels demonstrated enhanced stretchability and toughness compared to PVA/PAM‐IL and PVA/PAM‐Na (**Figure** **2**a,b). The strain at break of the ionogels increased, reaching a peak of 425% for PVA/PAM‐NaIL 2. Correspondingly, the calculated toughness, indicative of energy dissipation, reached its zenith at 20.36 MJ m^−3^ for PVA/PAM‐NaIL 2. PVA/PAM‐Na succumbed to a brittle fracture and failed to lift the weight. Conversely, the PVA/PAM‐NaIL 2 ionogel successfully lifted the weight without failure (Figure 2c). The PVA/PAM‐NaIL ionogels presented a strong fluorescence emission peak at ≈410 nm (blue‐emitting) when excited at wavelengths ranging from 380 to 400 nm (Figure 2d). After stopping ultraviolet (UV) lamp excitation, the afterglow lifetime was examined. The PVA/PAM‐IL ionogels readily generated a short afterglow of 56.9 ms due to the formation of small crystallites during the freeze‐thaw process. Clearly, the RTP lifetime experienced a significant enhancement in the PVA/PAM‐NaIL ionogels, with the most notable increase observed in PVA/PAM‐NaIL 2, which exhibited the longest lifetime of 112.4 ms (Figure 2e); interestingly, this trend aligns with the mechanical properties.

The evolution of the mechanical properties and RTP performance stems from the varied phase‐induced microstructures as a function of the composition, which can provide intuitive evidence from scanning electron microscopy (SEM) images. Pure PVA/PAM‐IL and PVA/PAM‐Na exhibited a uniform distribution of elements and smooth morphology, indicating a homogeneously dispersed network, as shown in Figure 2g and Figure S2 (Supporting Information). With increasing concentrations of CO_3_^2−^, a biphasic structure became apparent; one phase prominently displayed the polymer signal (polymer‐rich phase), while the other primarily showed the anion signal from the IL (IL‐rich phase). The polymer‐rich phases likely bridge the IL‐rich phases, creating a bicontinuous phase network that dissipates energy during deformation. However, the extensive phase separation observed in–PAM the NaIL 3 may have resulted in inadequate connectivity between the polymer‐ and IL‐rich phases. This lack of connectivity can significantly compromise the mechanical properties of the ionogels. This result was further validated in terms of ionic conductivity, as the ionic conductivity of PVA/PAM‐NaIL 2 was lower than that of PVA/PAM‐IL but significantly higher than that of PVA/PAM‐NaIL 3 (Figure S3, Supporting Information) because the largely disconnected aggregates increase the percolation threshold for electronic conduction.^[^ ^14^ ^]^ In contrast, we speculate that overhigh CO_3_^2−^ concentration would make the rearrangement and aggregation of molecular chains during the salting‐out process more chaotic, leading to increased structural disorder, which affects the mechanical properties and reduces the phosphorescence lifetime. Additionally, the RTP lifetime did not increase with increasing crystallinity, as seen from the X‐ray diffraction (XRD) patterns (Figure S4, Supporting Information), suggesting that the microphase‐separation‐mediated RTP mechanism was dominant rather than crystallization for the RTP enhancement of the PVA/PAM‐NaIL ionogels. PVA/PAM‐Na, with the highest crystallinity, exhibited a very short afterglow lifetime, similar to PVA/PAM‐IL, thus demonstrating that, beyond the restricted vibration, densely clustered electron‐rich groups (such as C═O and N─H) within the polymer‐rich phase significantly contribute to extended electron delocalization through space conjugation and lowered energy gaps for RTP emission.^[^ ^12^ ^]^ The stretchable PVA/PAM‐NaIL ionogels with ultralong phosphorescence are successfully fabricated. The afterglow was readily recognized by the naked eye (Figure 2h). The PVA/PAM‐NaIL 2 ionogels were used for subsequent investigations unless stated otherwise.

The polymer‐rich phase contributes to the ultralong RTP emission (Figure 2f),^[^ ^15^, ^16^ ^]^ whereas the IL‐rich phase provides effective ionic transport channels to enable electrical conductivity. As a result, a high conductivity of 8.4 mS cm^−1^ was achieved in PVA/PAM‐NaIL 2 ionogels (Figure S5, Supporting Information). The high ionic conductivity achieved while maintaining good mechanical properties was attributed to the decoupling effect of the unique microphase separation structure and other transport ions (i.e., Na^+^) introduced by the salting‐out process.

### 2.2 DFT Simulation of RTP Mechanism

**Figure 3:**
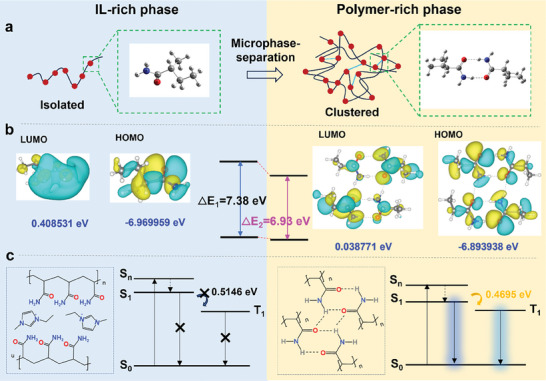
RTP mechanism of PVA/PAM ionogels. a) Schematic depiction of the amide bond transition from an isolated to a clustered state upon crystallization and subsequent microphase separation. b) HOMO and LUMO orbits. c) Proposed molecular interaction network of PAM molecular chain in IL‐rich and polymer‐rich phases and their respective photophysical characteristics.

As illustrated in **Figure** **3**a, ILs disrupt the inter‐ and intramolecular interactions, leading to a predominance of isolated molecular chains in the IL‐rich phase. Conversely, interactions involving the formation of hydrogen bonds between C═O and N─H moieties in the polymer‐rich phase readily induce clustered structures. The clustered structures facilitate extensive electron delocalization, resulting in a narrow bandgap. Initially, n–π* interactions contribute to the reduction of the bandgap, with n electrons originating from O and π electrons from a C═O double bond.^[^ ^17^ ^]^ The abundance of N atoms in the polymer‐rich phase was crucial for the RTP emission of the ionogels. Owing to the presence of multiple lone‐pair electrons on N and interactions with electron‐donating groups (─NH_2_), electrons can undergo not only n‐π* transitions but also π‐π* transitions.^[^ ^18^ ^]^ Density function theory (DFT) simulations supported the abovementioned results (Figure 3b,c). The results reveal that the clustered non‐traditional luminogens in the polymer‐rich phase possess a smaller HOMO/LUMO gap (ΔE_2_ = 6.93 eV) than that of isolated luminogens (ΔE_1_ = 7.38 eV). The intersystem crossing (ISC) process followed a HOMO‐to‐LUMO transition for RTP emission. In addition, the calculated △Est decreases from 0.5146 to 0.4695 eV, implying the ISC process is promoted by the microphase separation to enhance the RTP. Therefore, we divided the RTP mechanism of the PVA/PAM NaIL ionogels into two parts: i) formation of microphase separation‐mediated aggregation and ii) CTE in the polymer‐rich phase.

### 2.3 Full‐Color Phosphorescent Ionogels

**Figure 4:**
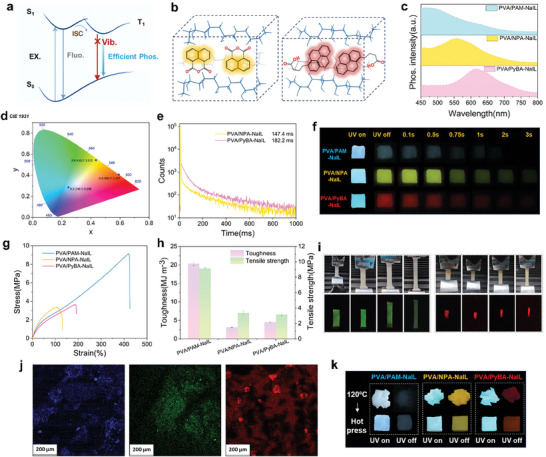
Full‐color RTP PVA‐based ionogels. a) Suppressing the vibration of the triplet of chromophores by crystallization and microphase separation to obtain long‐lived phosphorescence of PVA‐based ionogels. b) Schematic illustration of the restricted NPA (left) and PyBA (right) molecules in PVA/NPA‐NaIL and PVA/PyBA‐NaIL ionogels. c) Phosphorescence spectra of PVA/PAM‐NaIL, PVA/NPA‐NaIL, and PVA/PyBA‐NaIL ionogels excited by 365 nm, respectively. d) CIE chromaticity diagram of PVA/PAM‐NaIL, PVA/NPA‐NaIL, and PVA/PyBA‐NaIL ionogels excited by 365 nm. e) Lifetime decay curves of PVA/NPA‐NaIL and PVA/PyBA‐NaIL ionogels. f) Time‐dependent phosphorescence photographs of PVA/PAM‐NaIL, PVA/NPA‐NaIL, and PVA/PyBA‐NaIL taken after ceasing 365 nm irradiation. g) Stress–strain curves of PVA/PAM‐NaIL, PVA/NPA‐NaIL, and PVA/PyBA‐NaIL. h) Tensile stress and calculated toughness of PVA/PAM‐NaIL, PVA/NPA‐NaIL, and PVA/PyBA‐NaIL. Error bars represent mean ± standard deviation (*n* = 3). i) Photographs of PVA/NPA‐NaIL and PVA/PyBA‐NaIL ionogels during stretching on a universal testing machine taken under daylight and after ceasing 365 nm irradiation. j) CLSM images of PVA/PAM‐NaIL (left), PVA/NPA‐NaIL (middle), and PVA/PyBA‐NaIL (right) ionogels. k) Photographs of cut pieces of PVA/PAM‐NaIL, PVA/NPA‐NaIL, and PVA/PyBA‐NaIL ionogels before and after hot pressing under 365 nm irradiation, and ceasing of irradiation.

In addition to the unique CTE phenomenon resulting from the microphase‐separated structure of ionogels, the highly rigid polymer‐rich phase intrinsically benefits from the suppression of the non‐radiative relaxation pathway from T_1_ to S_0_ of chromophores because of its multiple hydrogen bonding interactions, which produce efficient RTP emission (**Figure** **4**a). Two common chromophores—1,8‐naphthalenedicarboxylic anhydride (NPA) and 1‐pyrenebutyric acid (PyBA)—were selected to fabricate green and red phosphorescent ionogels (Figure 1). The rigid environments of NPA and PyBA in the polymer‐rich phase are shown in Figure 4b. Similarly, the PVA/NPA‐NaIL and PVA/PyBA‐NaIL ionogels exhibited blue fluorescence with an emission centered at ≈410 nm when excited at wavelengths from 380 to 400 nm (Figure S6, Supporting Information). After 365 nm UV light excitation, the RTP emission centers of the obtained PVA/PAM‐NaIL, PVA/NPA‐NaIL, and PVA/PyBA‐NaIL ionogels were observed at 460, 555, and 615 nm, respectively (Figure 4c). The color coordinates of the phosphorescence of PVA/PAM‐NaIL, PVA/NPA‐NaIL, and PVA/PyBA‐NaIL ionogels correspond to (0.246, 0.285), (0.435, 0.543) and (0.592, 0.407) respectively, through the CIE (Commission International d’Éclairage) 1931 chromaticity coordinates (Figure 4d). In addition, the PVA/NPA‐NaIL and PVA/PyBA‐NaIL ionogels exhibit ultralong RTP lifetimes of 147.4 and 182.2 ms (Figure 4e). The full‐color afterglow of the PVA‐based ionogels is shown in Figure 4f.

The mechanical properties of PVA/NPA‐NaIL and PVA/PyBA‐NaIL were evaluated, revealing a decrease in tensile strength compared to that of PVA/PAM‐NaIL (Figure 4g,h). However, all the PVA‐based ionogels demonstrated significant elasticity (capable of stretching to over 100% before breaking) and moderate toughness for tangling, twisting, knotting, and stretching. As shown in Figure 4i, Figure S7, and Videos S1, S2 (Supporting Information), the PVA/NPA‐NaIL and PVA/PyBA‐NaIL ionogels maintained bright ultralong RTP under different strain conditions. Moreover, confocal laser scanning microscopy (CLSM) images of the PVA/PAM‐NaIL, PVA/NPA‐NaIL, and PVA/PyBA‐NaIL ionogels revealed that the luminescent regions within the ionogels were discontinuous (Figure 4j), further indicating the microphase‐separated structures of the PVA‐based ionogels.

The thermal properties of ionogels are considered important parameters for their processability. PVA/PAM‐NaIL is a solid‐like gel, considering its storage modulus (G′) exceeds its loss modulus (G″) in the frequency range of 0.1–100 Hz (Figure S8a, Supporting Information). With an increase in temperature, the crossover point at which G″ > G′ is present after 98 °C, after which the mixture flows readily (Figure S8b, Supporting Information). Therefore, the PVA/PAM‐NaIL, PVA/NPA‐NaIL, and PVA/PyBA‐NaIL ionogels exhibited excellent recyclability upon hot pressing. The full‐color afterglows can be easily recognized by the naked eye (Figure 4k), featuring not decayed ultralong lifetimes of 133.2, 179.0, and 133.1 ms, respectively (Figure S9, Supporting Information), after one cycle.

### 2.4 Electrical Sensing Properties

**Figure 5:**
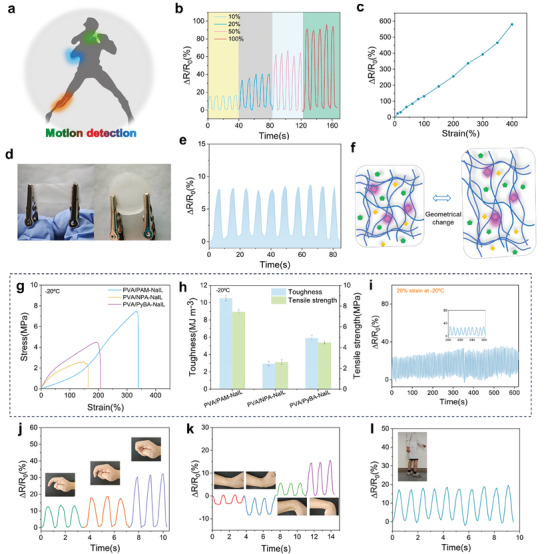
Sensing performance of the stretchable PVA/PAM‐NaIL ionogels. a) Ionic components within the PVA/PAM‐NaIL ionogel endow it with superior conductivity and the ability to monitor body motion. b) Relative change in resistance under repeated loading–unloading processes at a strain of 10%, 20%, 50%, and 100%, respectively. c) Relative change in resistance generated as a function of applied strains. d) Photographs of PVA/PAM‐NaIL ionogels undergoing tensile stress (left) and compressive stress (right) for electrical property evaluation. e) Relative change in resistance of PVA/PAM‐NaIL ionogel under consecutive compression. f) Proposed mechanism of the influence of external stimuli on the ionic conductivity of PVA/PAM‐NaIL ionogel. g) Stress–strain curves of PVA/PAM‐NaIL, PVA/NPA‐NaIL and PVA/PyBA‐NaIL ionogels at −20 °C. h) Tensile stress and calculated toughness of PVA/PAM‐NaIL, PVA/NPA‐NaIL and PVA/PyBA‐NaIL at −20 °C. Error bars represent mean ± standard deviation (*n* = 3). i) Relative resistance variation during cyclical loading and unloading at a 20% strain at −20 °C. Real‐time monitoring of j) finger movements, k) wrist bending, and l) movement characteristics during walking. The inset shows PVA/PAM‐NaIL ionogel attached to the knuckle, wrist, and foot.

We assume that using the prepared PVA‐based ionogels as an i‐Skin, the electrical signals generated by deformation can be utilized for movement monitoring, and the distinct phosphorescent colors can serve as indicators of the specific areas where movement occurs, as illustrated in **Figure** **5**a. The sensing performance of the PVA/PAM‐NaIL ionogel in response to mechanical deformation was evaluated (Figure 5d). The successive cyclic stretching and releasing tests were performed to observe the relative change in electrical resistance (ΔR/R_0_) with increased strains (Figure 5b). The results showed that the resistance of the PVA/PAM‐NaIL ionogel could accurately and reversibly respond to various strains (10%–100%). Additionally, a strong linear relationship was observed between the strain and electrical signals, with a correlation coefficient of 0.995 (Figure 5c). The gauge factor, defined as (ΔR/R_0_)/ɛ, is measured to be 1.36. The cyclic stretching and releasing test during 100 uninterrupted tests at a strain of 10% reveals the high reliability and durability of the ionogel, as illustrated in Figure S10 (Supporting Information). The PVA/PAM‐NaIL ionogel was also employed for compression‐sensing performance testing. As shown in Figure 5e, it exhibits stable and reliable relative resistance changes under compression. A force applied to the ionogels caused a transient stress distribution within them, leading to geometrical changes (Figure 5f). These changes can result in charge redistribution, which, in turn, causes a change in the ionogel resistance.

Exploiting the inherently low freezing point of ILs, PVA‐based ionogels can function effectively in subzero environments. Tensile tests were conducted on PVA/PAM‐NaIL, PVA/NPA‐NaIL, and PVA/PyBA‐NaIL ionogels at −20 °C. The results show a slight decrease in the stretchability of the PVA/PAM‐NaIL ionogel, whereas the PVA/NPA‐NaIL and PVA/PyBA‐NaIL ionogels exhibit increased stretchability (Figures 4g and 5g). Overall, the ionogels maintained favorable mechanical properties after 24 h of storage at −20 °C, as seen in Figure 5h and Figure S11 (Supporting Information). The PVA/PAM‐NaIL ionogel demonstrated a tensile strength of ≈8.94 MPa and an elongation at break of ≈340%. The sensing abilities of PVA/PAM‐NaIL ionogels at −20 °C and mild conditions were compared; the ΔR/R_0_ signals remained relatively stable from the results shown in Figure 5i.

We also performed on‐skin strain‐sensing experiments to evaluate whether the prepared ionogels were suitable candidates for mimicking human physiological movements and their excellent sensing capabilities. As shown in Figure 5j–l, the PVA/PAM‐NaIL ionogels readily deform by finger flexion and other body movements. They can detect changes in the relative resistance corresponding to varying degrees of finger flexion (30°, 60°, and 90°) and body movements such as wrist flexion (Figure 5k) and walking (Figure 5l). Stable electrical signals were obtained under different deformations, demonstrating the excellent reliability of the ionogels for smart mechanotransduction devices.

### 2.5 Potential Applications

**Figure 6:**
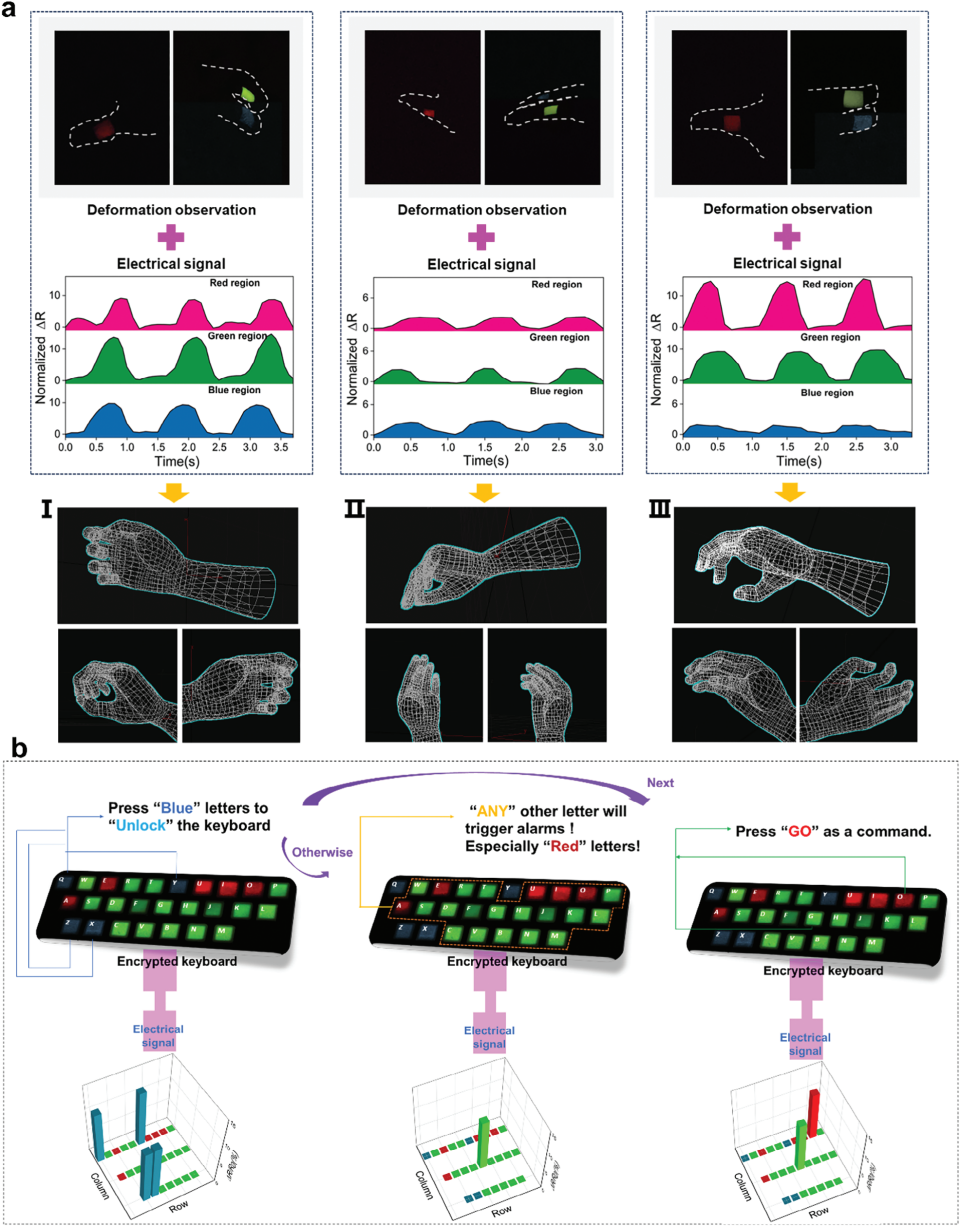
Applications of PVA‐based ionogels. a) High‐precision simulations of robotic hand grabbing states through a synergistic dual‐mode prediction strategy, which integrates visual observations of deformation across various phosphorescent color regions with the corresponding electrical signal fluctuations during a grasping action. b) Multicolor afterglow display codes the keyboard after switching off the UV lamp, coupled with the electrical signal variation upon typing, a highly secure information encryption and transmission system is constructed.

In previous studies, the movement assessment of soft robots was limited to electrical signals, resulting in low accuracy.^[^ ^19^ ^]^ Herein, a novel human‐machine interaction mode is constructed by combining full‐color RTP and mechano‐transduction ability. Stretchable PVA‐based ionogels with different phosphorescent colors were placed at the joints of the robotic hand, as shown in Figure S12 (Supporting Information). Different phosphorescent colors can provide additional positional information, such as red for the thumb area, green for the index finger, and blue for the middle finger. Gesture information of the robot hand can be preliminarily obtained by recognizing the colorful image of the “fingers” during movement. As shown in **Figure** **6**a, based on the deformation of the ionogels in various directions, a picture of the different gestures of the robotic hand was sketched. Meanwhile, the PVA‐based ionogels provided real‐time motion feedback of the robotic hand during deformation (i.e., large fluctuations for Gesture I, smaller fluctuations for Gesture II, and largest fluctuations in the red region for Gesture III). Based on the above results, we infer that Gesture I corresponds to a ring shape composed of three fingers, Gesture II resembles a duck's beak, and Gesture III involves two fingers bent inward. The objects associated with these gestures may be balls, paper, or cylinders. Notably, the ultralong afterglow and bright RTP color of the ionogels can be easily recognized by the naked eye, and, when combined with electrical signals, users can recognize the body language information of soft robots more intuitively and accurately.

Furthermore, we investigate their potential in the field of security defense through multicolor afterglow display‐assisted data encryption and transmission. As shown in Figure 6b, a keyboard made of stretchable full‐color phosphorescent PVA‐based ionogels was fabricated. The keyboard switches to the encrypted mode when the UV excitation ceases, with specific information encoded by varying the RTP colors. The relative changes in electrical resistance when a key is pressed serve as transfer signals. The keyboard could only be unlocked by correctly pressing the keys marked with “blue” phosphorescent letters, which generated the necessary electrical signals; otherwise, it is deemed an accidental touch that prevents the transmission of information. The vowels A, E, I, O, and U are displayed in “red” phosphorescence on the keyboard due to their importance in spelling words. If electrical signals are detected from these keys before the keyboard is unlocked, an alarm is triggered. Stretchable ionogels facilitate dynamic interaction and visual feedback, combining their unique antibacterial and flame‐retardant properties (Figures S13, S14, Supporting Information), presenting an innovative approach for developing next‐generation optoelectronics.

## 3 Conclusion

In summary, we discovered that salting‐out ions (CO_3_^2−^) can effectively interact with PVA chains to push the ionic liquids past the gel phase boundary, resulting in microphase separation within the ionogels. In this structure, the IL‐rich (soft) phase facilitates stretching and ionic conduction, whereas the polymer‐rich (stiff) phase aids energy dissipation and RTP. This approach allowed for the synergistic enhancement of the mechanical properties and RTP emission of the ionogels. The resulting PVA/PAM‐NaIL ionogels exhibit high stretchability (≈400% strain), toughness (≈20 MJ m^−3^), conductivity (8.4 mS cm^−1^), and an ultralong afterglow lifetime (112.4 ms). Additionally, we incorporated a range of chromophores, such as NPA and PyBA, to create color‐tunable phosphorescent ionogels with afterglow lifetimes of 147.4 and 182.2 ms, respectively. The PVA‐based ionogels can detect various stimuli and generate output electrical signals, even at −20 °C. By leveraging the observable full‐color RTP and real‐time electrical signals produced by stimuli such as stretching and pressing, we developed the i‐Skin and encrypted keyboards with dynamic interaction and visual feedback capabilities. The straightforward fabrication process, wide tunability, and environmental tolerance of ionogels are expected to enable the design of multifunctional soft machines with applications in the biomedical and engineering fields.

## 4 Experimental Section

### Reagents and Materials

Polyvinyl alcohol (PVA‐1799), polyacrylamide (PAM), N, N‐dimethylformamide (DMF), and dimethyl sulfoxide (DMSO) were purchased from Chengdu Chron Chemical Co., Ltd. Anhydrous sodium carbonate (Na_2_CO_3_, 99.5%), 1‐ethyl‐3‐methylimidazolium chloride, and 1,8‐naphthalenedicarboxylic anhydride (NPA) were purchased from Macklin Biochemical Co. Ltd. 1‐Pyrenebutyric acid (PyBA) was purchased from Shanghai Haohong Scientific Co., Ltd. All chemical reagents were used without further purification.

### Preparation of PVA/PAM‐NaIL Ionogels

PVA (1.5 g) was dissolved in deionized water (8.5 mL) and continuously stirred at 95 °C to obtain a homogeneous solution. Subsequently, 1.6 g of 1‐ethyl‐3‐methylimidazolium chloride and 2 mL of a PAM solution with a 5% mass fraction were introduced sequentially. Next, 0, 0.1, 0.3, and 0.5 g of Na_2_CO_3_ were added separately. Once a completely transparent solution was obtained, the mixture was decanted into a tetrafluoroethylene mold and subjected to freezing at −20 °C for 12 h, followed by a 6 h drying process at 60 °C to obtain PVA/PAM‐NaIL ionogels.

### Preparation of PVA/NPA‐NaIL and PVA/PyBA‐NaIL Ionogels

*P*VA/NPA‐NaIL and PVA/PyBA‐NaIL ionogels were prepared using a method similar to that for PVA/PAM‐NaIL, with 1.6 g of 1‐ethyl‐3‐methylimidazolium chloride and 0.3 g Na_2_CO_3_. In addition, 0.1 g of NPA in 2 mL of DMSO and 0.1 g of PyBA in 2 mL of DMF were added as chromophores.

### Characterizations

The PL spectra and time‐resolved phosphorescence decay curves were recorded using an FLS‐980 photoluminescent spectrometer (Edinburgh Instruments, UK). The morphologies of the ionogels were examined by scanning electron microscopy (SEM; ZEISS GeminiSEM 360) at a voltage of 10 kV. X‐ray diffraction (XRD) was performed using a RIGAKU Ultima IV instrument. Confocal laser scanning microscopy (CLSM) was performed using a Leica Stellaris 5 platform. The rheological properties of ionogels were assessed over a frequency range spanning from 0.1 to 100 Hz at 25 °C and further explored across a temperature gradient from 20 to 100 °C under a heating rate of 5 °C min^−1^ utilizing a dynamic mechanical analyzer (NETZSCH Kinexus Lab+).

### Tensile Testing

Tensile characteristics were evaluated using a universal testing machine (UTM‐4103, China). The ionogels were precisely sectioned into rectangular specimens measuring 50 × 10 × 2 mm and subjected to a strain rate of 10 mm min^−1^. Initially, the sample length between grips was 30 mm. The tensile modulus of the ionogel was determined by analyzing the gradient of the stress–strain curve within the 5–15% strain range. The toughness of the ionogel is represented by the area under the stress–strain curve. Tensile tests were conducted on specimens stored at −20 °C for over 24 h to assess the mechanical characteristics of ionogels at low temperatures.

### Electronic Properties

The electrical signals of the PVA‐based ionogels under external stimuli were recorded using an electrochemical workstation (CHI760E, Chenhua, China) with an applied voltage of 1 V and a frequency of 1 kHz. To continuously measure resistance during stretching, the ionogels were fixed to a homemade stretching station with electrical leads at both ends. The tensile strain was calculated from the initial and final lengths using a ruler. The conductivities of the ionogels were calculated according to Equation (1), where R is the resistance, d is the length, and A is the cross‐sectional area of the ionogel.^[^ ^20^ ^]^

(1) $$\sigma =d/\left(R\times A\right)$$

### Computational Details

Theoretical calculations were performed using the Gaussian 16 program suite.^[^ ^21^ ^]^ The structures of the model molecules were fully optimized at the B3LYP‐D3BJ/TZVP level of theory. The vibrational frequencies of the optimized structures were measured at the same level. The structures were characterized as a local energy minimum on the potential energy surface by verifying that all vibrational frequencies were real. The molecular orbital levels of the studied compounds, including the highest occupied molecular orbital (HOMO) and lowest unoccupied molecular orbital (LUMO), were investigated. The VESTA program was used to plot color‐filled isosurface graphs to visualize the molecular orbitals.^[^ ^22^ ^]^ The energy levels of the lowest singlet (S_1_) and lowest triplet (T_1_) excited states were calculated by the vertical excitation of the optimized structures using the TD B3LYP‐D3BJ/TZVP level of theory.

### Antibacterial Performance Test

The agar medium (5 g peptone, 3 g beef extract, 5 g NaCl, and 15 g agar, pH 7.0) was sterilized and transferred to a sterile Petri dish for solidification. Then, 0.1 mL Escherichia coli suspension (10^6^–10^7^ CFU mL^−1^) was evenly spread on the agar plate and incubated upside down in a 37 °C culture room for 24 h. Finally, the inhibition ring was observed.

### Statistical Analysis

Origin 2021 software was used to assess the statistical significance of all comparative studies. Data are presented as the mean ± SD; *n* = 3 independent experiments.

## Conflict of Interest

The authors declare no conflict of interest.

## Supporting information



Supporting Information

Supplemental Video 1

Supplemental Video 2

## Data Availability

The data that support the findings of this study are available on request from the corresponding author. The data are not publicly available due to privacy or ethical restrictions.
