# Supplementary material for: Stretchable Full‐Color Phosphorescent PVA‐Based Ionogels for Multimodal Sensing‐Visual Integration Applications
Source: Adv Sci (Weinh). 2024 Dec 12;12(5):2411229. doi: 10.1002/advs.202411229 (PMC11792044; doi:10.1002/advs.202411229)
Supplement: Supplementary file 1 — Supporting Information [file ADVS-12-2411229-s003.docx]

Supporting Information

Stretchable Full-Color Phosphorescent PVA-based Ionogels for Multimodal Sensing-Visual Integration Applications

*Xuefeng Wei, Zexi Gou, Jianting Ye, Jianwei Zhao, Lei Yang, Linbo Zhang*, Kun Zhang, Ruonan Jia**

**This file includes:**

Supplementary Figure 1 to 15

**Other Supplementary Materials for this manuscript include the following:**

Supplementary Movie 1 to 2


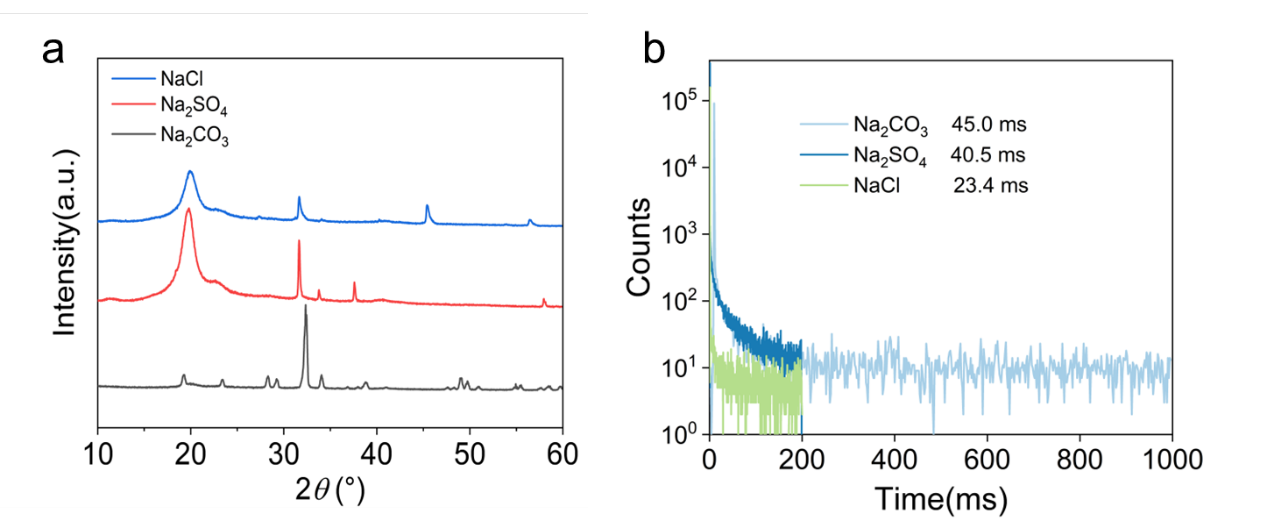


**Figure S1. Effect of various inorganic salts on structure and RTP properties. a** XRD patterns of PVA/PAM ionogels with different inorganic salts. **b** Lifetime decay curves of PVA/PAM ionogels with different inorganic salts.


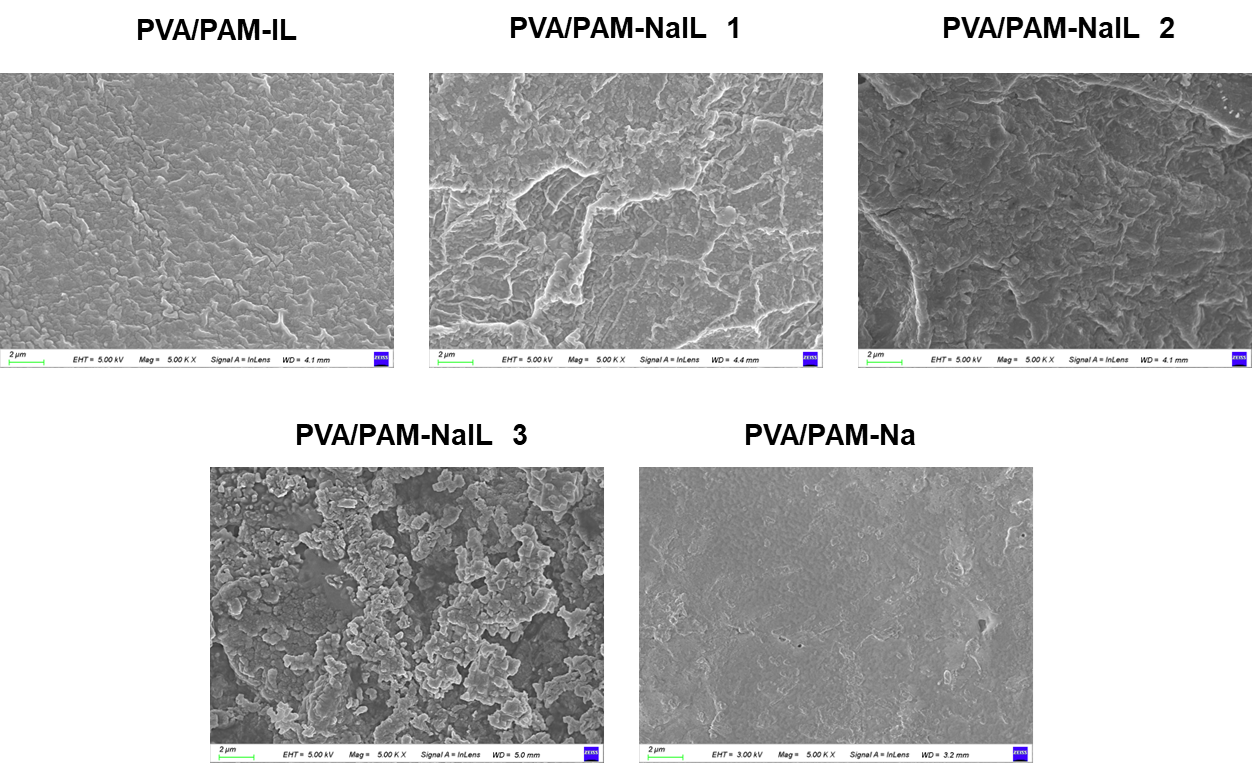


**Figure S2. Morphology of PVA/PAM ionogels.** SEM images of PVA/PAM-IL, PVA/PAM-NaIL and PVA/PAM-Na.


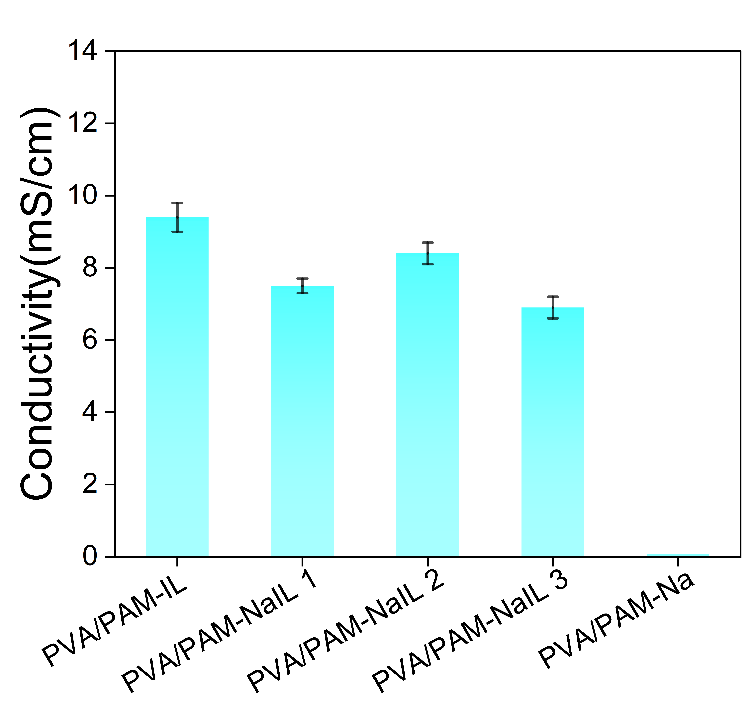


**Figure S3. Conductivity of** **PVA/PAM ionogels.** The electrical conductivity of PVA/PAM ionogels with different formulations. Error bars represent mean ± standard deviation (n =3).


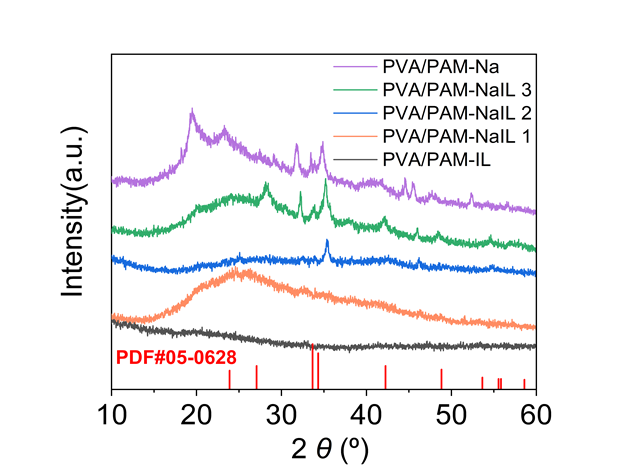


**Figure S4. Crystalline structure.** XRD patterns of PVA/PAM ionogels and diffraction peaks of Na_2_CO_3_ (PDF#05-0628).


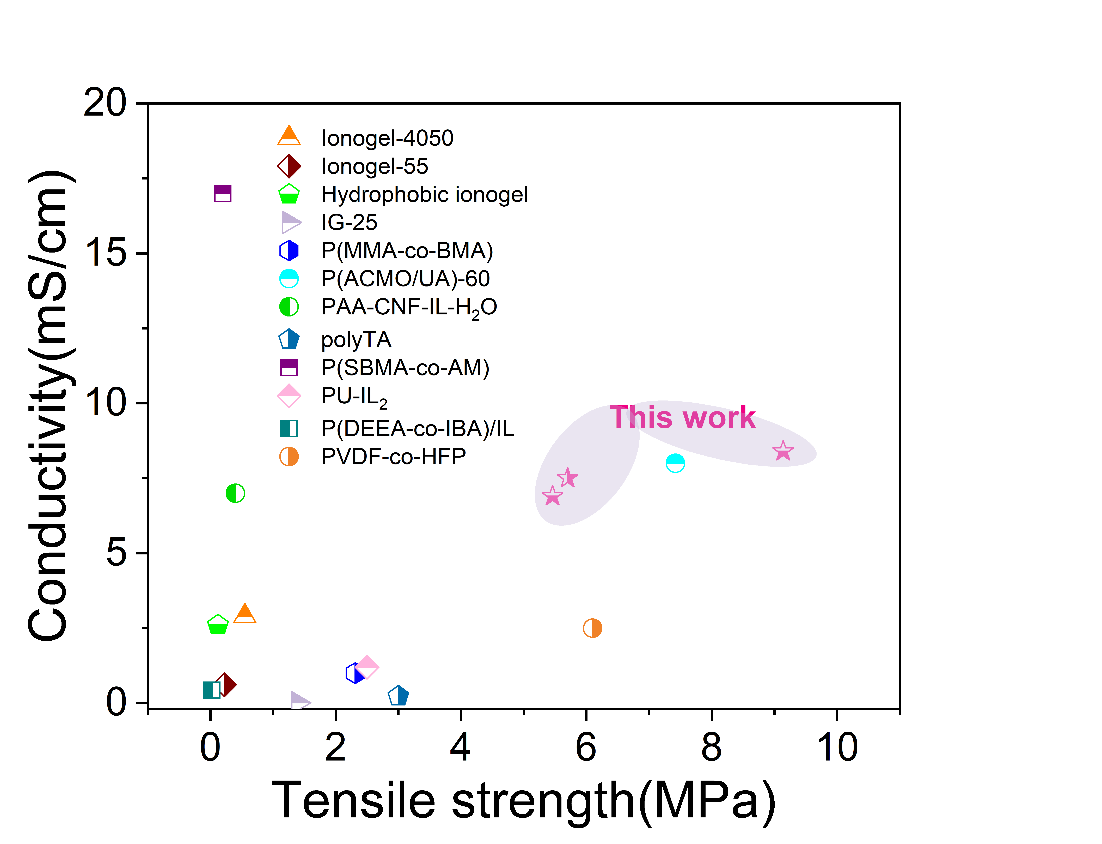


**Figure S5. Superior conductivity of the prepared PVA/PAM ionogels.** Comparison plots of the ionogels in this article with other recently reported works by tensile strength versus conductivity. ^[1-12]^


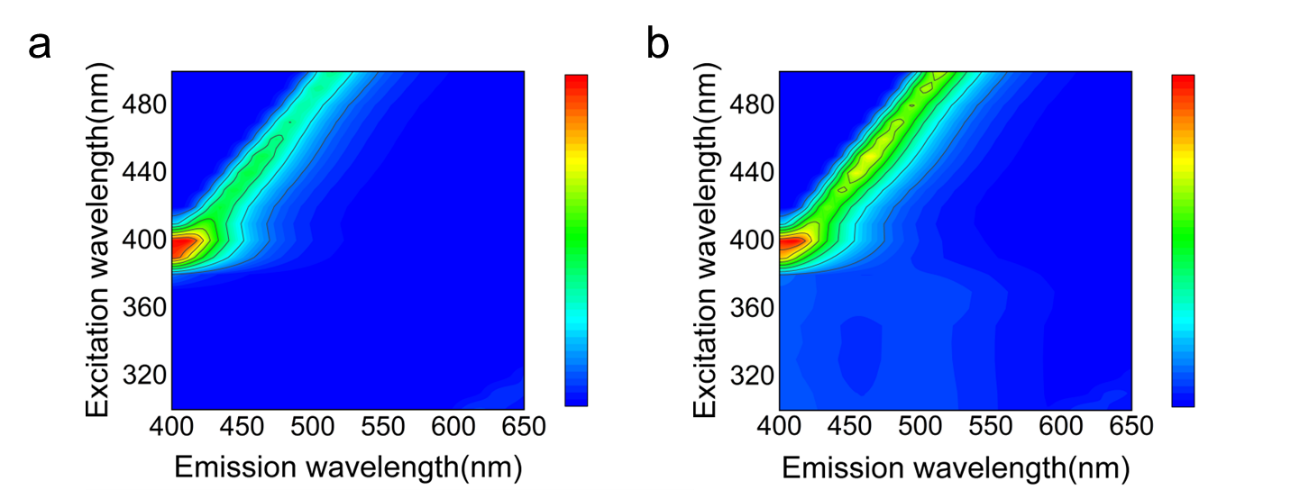


**Figure S6. Excitation fluorescence mapping of PVA-based ionogels with different chromophore. a** PVA/NPA-NaIL ionogel. **b** PVA/PyBA-NaIL ionogel.


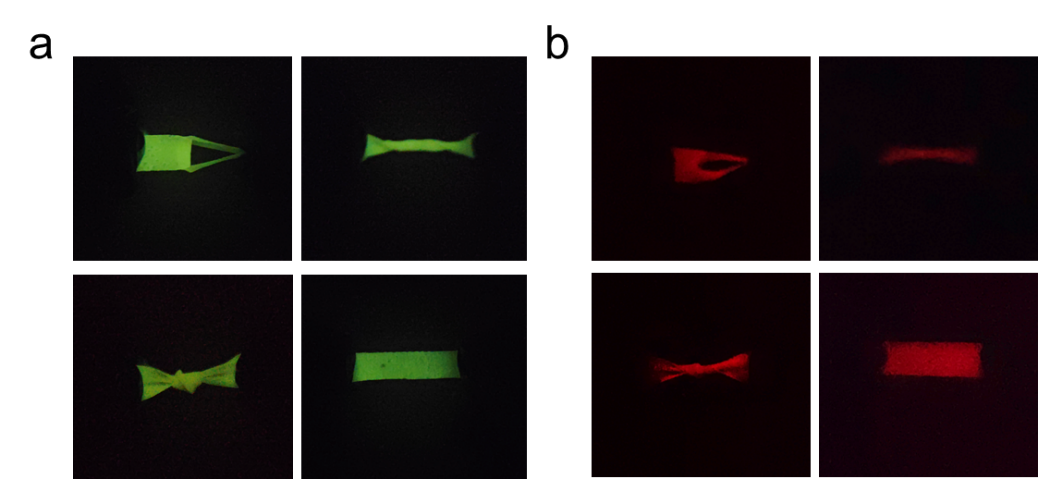


**Figure S7. The RTP photographs of the PVA-based ionogels in tangled, twisted, knotted and stretched states. a** PVA/NPA-NaIL ionogel. **b** PVA/PyBA-NaIL.


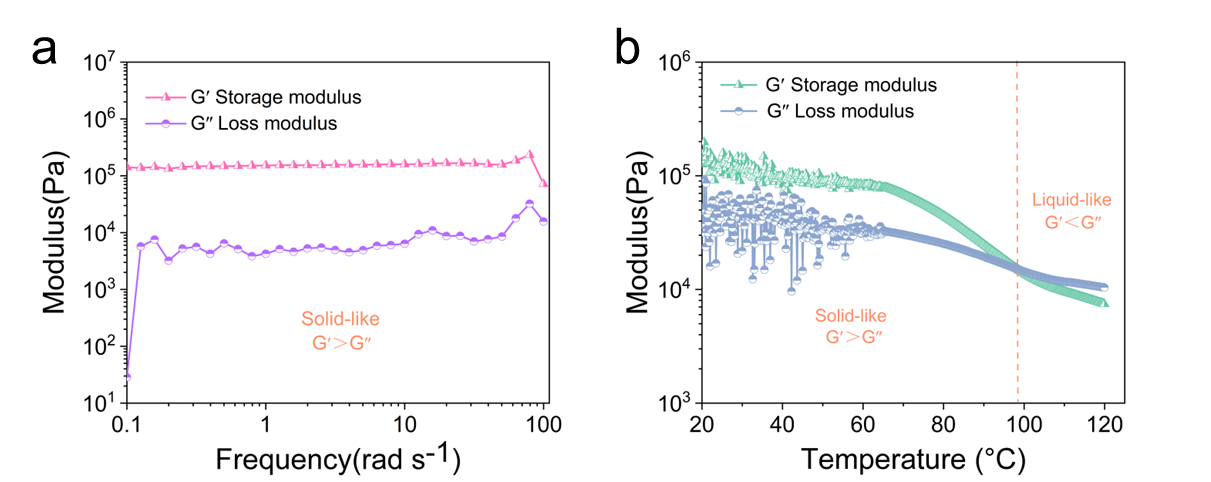


**Figure S8. Dynamic mechanical analysis. a** Modulus profile of PVA/PAM-NaIL 2 as a function of frequency. **b** Modulus profile of PVA/PAM-NaIL 2 as a function of temperature.


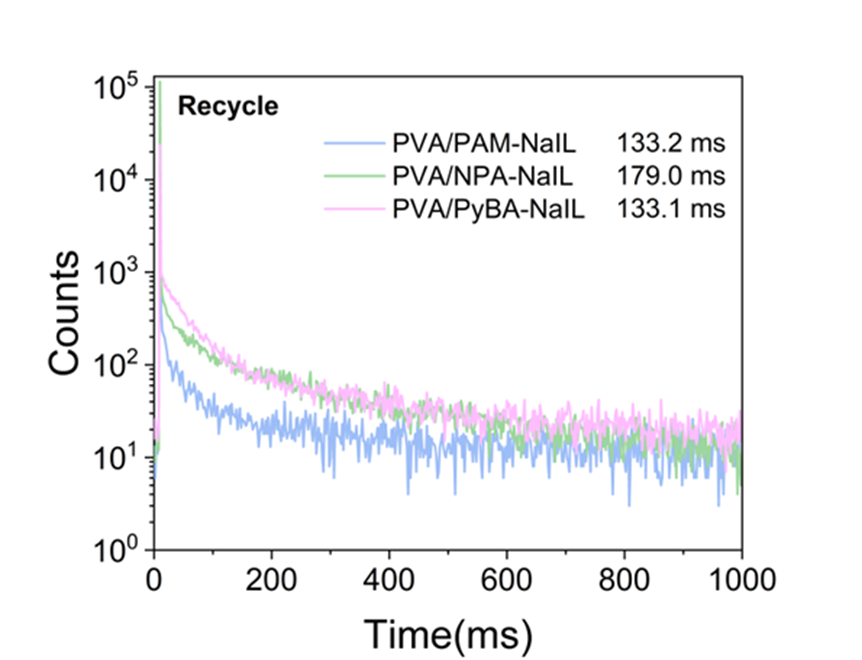


**Figure S9. Cyclic stability of phosphorescence lifetime.** Lifetime decay curves of cyclically obtained PVA/PAM-NaIL, PVA/NPA-NaIL and PVA/PyBA-NaIL ionogels.


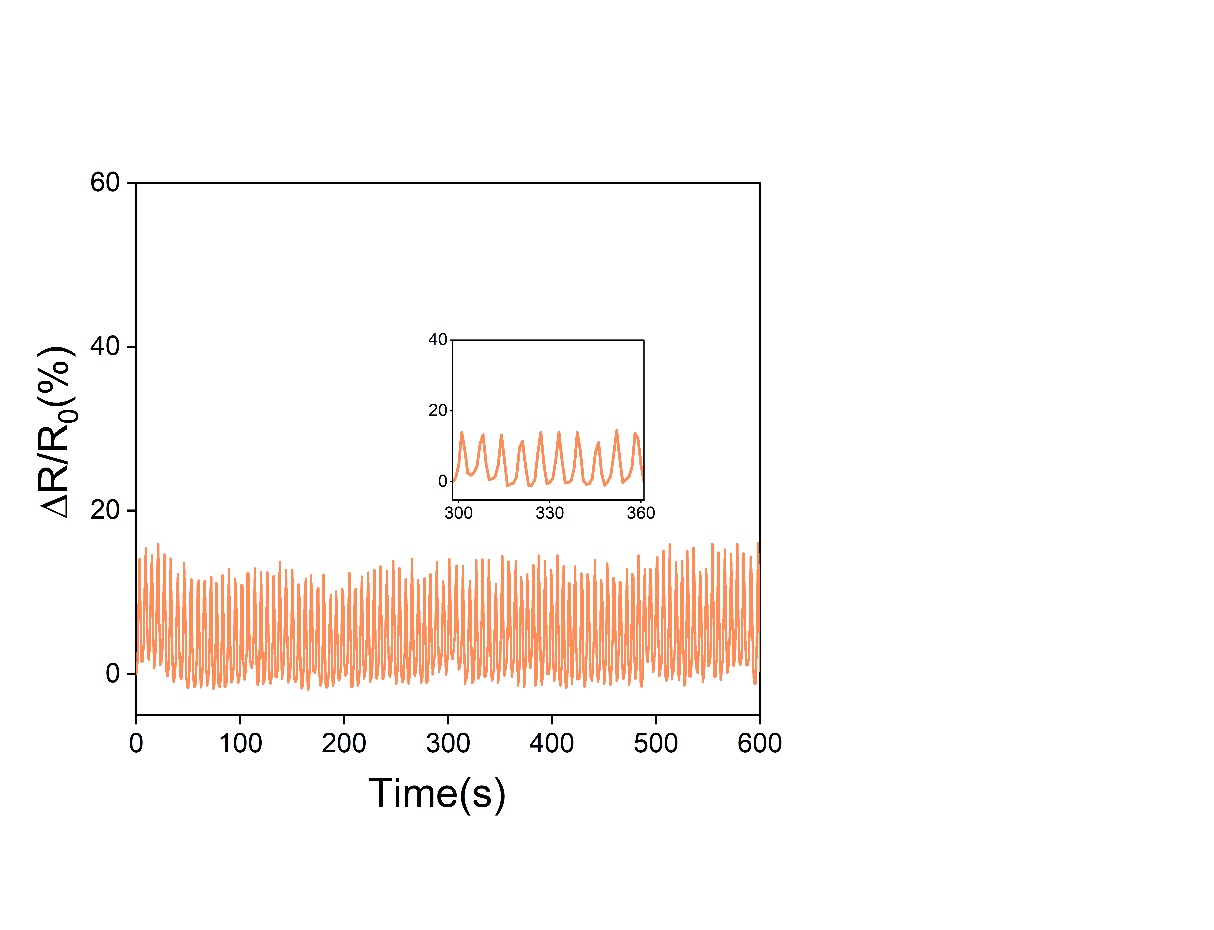


**Figure S10. Electric property.** Relative change in resistance under repeated loading–unloading processes at a strain of 10% for uninterrupted 100 cycles.


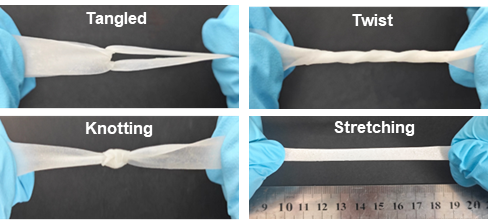


**Figure S11. Anti-freezing performance.** Photographs of PVA/PAM-NaIL ionogels in tangled, twisted, knotted and stretched states after storage at -20ºC for 24 h.


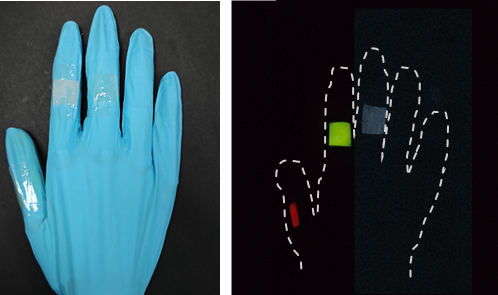


**Figure S12. Simulations of robotic hand.** PVA-based ionogels at the joints of robot hand (left), and its RTP image (right).


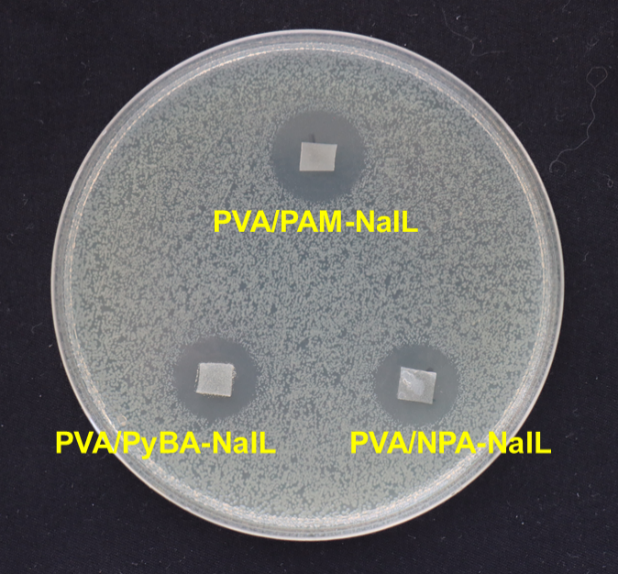


**Figure S13. Antibacterial properties.** Inhibition ring experiment of PVA/PAM-NaIL, PVA/NPA-NaIL and PVA/PyBA-NaIL ionogels towards *E. coli*.


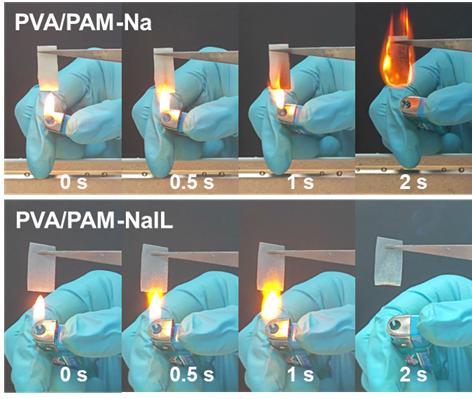


**Figure S14. Flame-retardant properties.** Combustion process of PVA/PAM-Na and PVA/PAM-NaIL at different times.


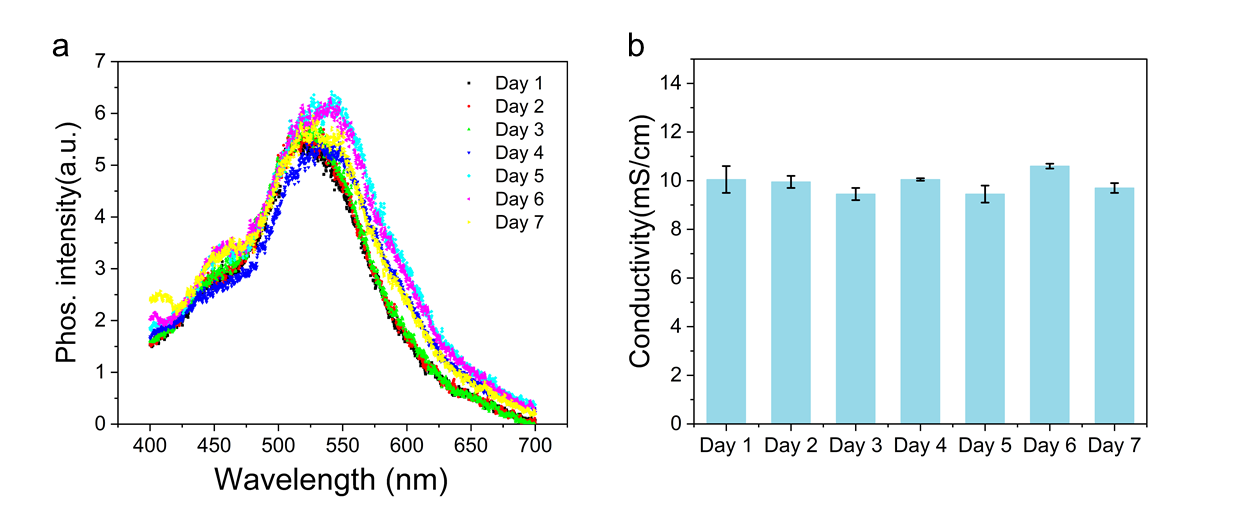


**Figure S15. Long-term stability.** The phosphorescence intensity (a) and conductivity (b) of ionogels kept in dry air for different times.

Video S1. The strechability and RTP performance of PVA/NPA-NaIL ionogel.

Video S2. The strechability and RTP performance of PVA/PyBA-NaIL ionogel.

**Reference:**

[1] L. Xu, Z. Huang, Z. Deng, Z. Du, T. L. Sun, Z.-H. Guo, K. Yue, *Adv. Mater.* **2021**, 33, 2105306.

[2] L. Sun, H. Huang, Q. Ding, Y. Guo, W. Sun, Z. Wu, M. Qin, Q. Guan, Z. You, *Adv. Fiber Mater.* **2022**, 4, 98-107.

[3] J. Wei, Y. Zheng, T. Chen, *Mater. Horiz.* **2021**, 8, 2761-2770.

[4] Z. Yu, P. Wu, *Adv. Funct. Mater.* **2021**, 31, 2107226.

[5] J. Lan, Y. Li, B. Yan, C. Yin, R. Ran, L.-Y. Shi, *ACS Appl. Mater. Interfaces*. **2020**, 12, 37597-37606.

[6] M. Zhang, X. Tao, R. Yu, Y. He, X. Li, X. Chen, W. Huang, *J. Mater. Chem. A*. **2022**, 10, 12005-12015.

[7] Y. Ye, H. Oguzlu, J. Zhu, P. Zhu, P. Yang, Y. Zhu, Z. Wan, O. J. Rojas, F. Jiang, *Adv. Funct. Mater.* **2023**, 33, 2209787.

[8] Y. Wang, S. Sun, P. Wu, *Adv. Funct. Mater.* **2021**, 31, 2101494.

[9] L. Zhao, B. Wang, Z. Mao, X. Sui, X. Feng, *Chem. Eng. J.* **2022**, 433, 133500.

[10] T. Li, Y. Wang, S. Li, X. Liu, J. Sun, *Adv. Mater.* **2020**, 32, 2002706.

[11] H. Wang, Y. Mao, D. Ji, L. Wang, L. Wang, J. Chen, X. Chang, Y. Zhu, *Chem. Eng. J.* **2023**, 471, 144674.

[12] W. Zhan, H. Zhang, X. Lyu, Z.-Z. Luo, Y. Yu, Z. Zou, *Science China-Materials*. **2023**, 66, 1539-1550.
